# Correspondence on “Salivary biomarkers in breast cancer diagnosis: A systematic review and diagnostic meta‐analysis”

**DOI:** 10.1002/cam4.6353

**Published:** 2023-09-27

**Authors:** Niloofar Dehdari Ebrahimi, Alireza Ahmadkhani, Ehsan Taherifard, Erfan Taherifard, Alireza Sadeghi

**Affiliations:** ^1^ School of Medicine Shiraz University of Medical Sciences Shiraz Iran

We have read the article “Salivary biomarkers in breast cancer diagnosis: A systematic review and diagnostic meta‐analysis” with great interest.^1^ The article presents a promising idea and concludes that salivary biomarkers are a promising noninvasive method for differentiating patients with breast cancer from healthy individuals. Future implications of the findings are of great interest for policy makers and individuals. However, we have identified some limitations in the methodology of the study that need to be addressed.

Firstly, the eligibility criteria used in the study do not provide enough details about which salivary biomarkers were investigated. The article only mentions the measurement methods and types of biomarkers (proteomics, metabolomics, transcriptomics, and reagent‐free biophotonic), but does not specify the actual biomarkers studied (e.g., c‐erbB‐2, vascular endothelial growth factor, epidermal growth factor, etc.). Providing this information would be useful for readers who want to understand the specific biomarkers that showed promise in the study.

Secondly, the study performed subgroup analyses based only on the method of measurement and the type of biomarker. However, pooling the data on different index tests (i.e., salivary biomarkers) in a meta‐analysis of diagnostic test accuracy studies, if not accompanied with further subgroup analyses, is prone to bias and confusion of the readers.

Thirdly, the study did not take the heterogeneity into account when evaluating the publication bias. The examination of funnel plots frequently reveals a proclivity for smaller studies to report effect sizes that differ from, and are often more inflated than, those reported by larger studies. This trend can be attributed to several underlying factors; however, between‐study heterogeneity and publication bias are the two most prevalent explanations. Tests for funnel‐plot asymmetry have been crafted to discern the presence of asymmetry in a given funnel plot. These tests, sometimes dubbed “tests of publication bias,” may be at risk of being misleading, as the existence of an asymmetry in a funnel plot is not necessarily caused by publication bias.^2^ The presence of between‐study heterogeneity bears the potential to impinge upon the symmetry of a funnel plot, thereby casting an impact on any statistical approach that relies on the evaluation of that symmetry.

Also, it is commonly believed that tests for funnel plot asymmetry (including the Begg, Egger, Harbord, and Peters tests) designed primarily for use in reviews of randomized trials and should not be used in systematic reviews of test accuracy. Therefore, Cochrane handbook for systematic reviews of diagnostic test accuracy recommend utilizing more appropriate methods that were developed for detecting funnel plot asymmetry in systematic reviews of test accuracy.^3^

Lastly, when evaluating the accuracy of a test to distinguish between healthy and ill individuals, it is important to consider other conditions that may cause positive and negative values. For example, in this study, the authors conclude that salivary biomarkers have the potential to accurately differentiate patients with breast cancer from healthy controls while ignoring the effect of other cancers on the same tests.^4^ In addition, the study fails to describe rigid eligibility criteria to exclude studies that have patients with distant metastases or combined primary cancer of breast and other organs included.

## AUTHOR CONTRIBUTIONS

**Niloofar Dehdari Ebrahimi:** Writing – original draft (equal); writing – review and editing (equal). **Alireza Ahmadkhani:** Writing – original draft (equal). **Ehsan Taherifard:** Writing – review and editing (equal). **Erfan Taherifard:** Writing – review and editing (equal). **Alireza Sadeghi:** Conceptualization (lead).

## CONFLICT OF INTEREST STATEMENT

The authors declare that they have no competing interest.
